# Targeted rescue of cancer-associated IDH1 mutant activity using an engineered synthetic antibody

**DOI:** 10.1038/s41598-017-00728-1

**Published:** 2017-04-03

**Authors:** Shahir S. Rizk, Somnath Mukherjee, Akiko Koide, Shohei Koide, Anthony A. Kossiakoff

**Affiliations:** 1 Department of Chemistry and Biochemistry, Indiana University South Bend, Department of Biochemistry and Molecular Biology, Indiana University School of Medicine, South Bend, Indiana USA; 2 Department of Biochemistry and Molecular Biology, University of Chicago, Chicago, Illinois USA; 3 Perlmutter Cancer Cener, New York University Langone Medical Center, New York, USA; 4 Department of Medicine, New York University School of Medicine, New York, USA; 5 Department of Biochemistry and Molecular Pharmacology, New York University School of Medicine, New York, USA

## Abstract

We have utilized a high-diversity phage display library to engineer antibody fragments (Fabs) that can modulate the activity of the enzyme isocitrate dehydrogenase 1 (IDH1). We show that a conformation-specific Fab can reactivate an IDH1 mutant associated with brain tumors. The results show that this strategy is a first step towards developing “activator drugs” for a large number of genetic disorders where mutations have disrupted protein function.

## Introduction

**Figure 1:**
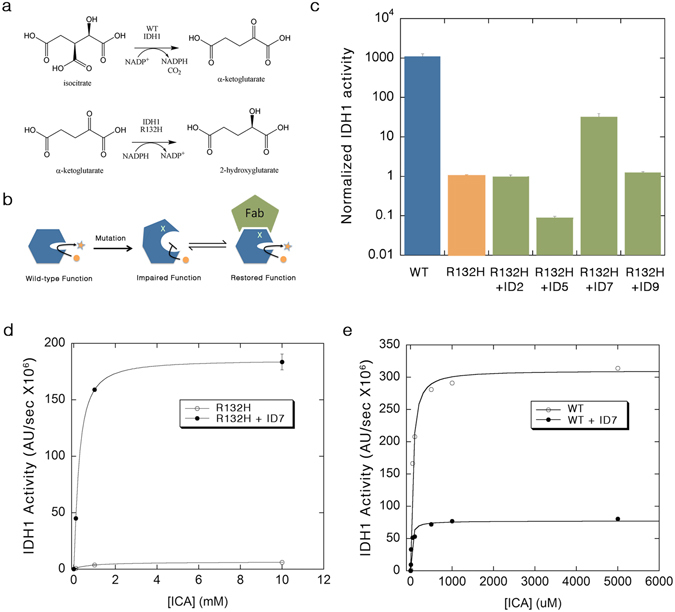
Rescuing the function of IDH1 mutant. (**a**) IDH1 catalyzes the NADP-dependent conversion of isocitrate to α–ketoglutarate. Mutations at R132 result in a neomorphic activity where NADPH is oxidized, and the oncometabolite 2-HG is produced. (**b**) A mutation (indicated by X) disrupts the conformation of the protein and results in an inactive enzyme. An engineered conformation-specific Fab that recognizes the active form of the enzyme can be used to reactivate the mutant enzyme by forcing it to adopt an active form. (**c**) WT IDH1 activity (blue) was compared to the activity of the IDH1 R132H mutant at 10 mM isocitrate in the absence (orange) or presence of excess Fabs (green). Fab ID7 results in a 30-fold enhancement of mutant activity (R132H activity set to 1). (**d**) The activity of the R132H mutant in the absence (open circles) and presence of ID7 (closed circles) was determined as a function of increasing isocitrate (ICA) concentrations. (**e**) In the presence of ID7 (closed circles), WT IDH1 shows a decrease in activity as well as a lower *K*_M_ value (see Table 1).

We present an approach for the design of engineered affinity reagents that can modulate enzyme activity and rescue the function of a tumor-associated mutant enzyme. Isocitrate dehydrogenase 1 (IDH1) converts isocitrate (ICA) to α–ketoglutarate (α-KG) by the reduction of NADP to NADPH^1^. Mutations at position R132 have been found in a large number of tumors, most prominently in up to 80% of low-grade gliomas, which typically progress to a fatal glioblastomas^2^. The most common mutant, R132H, was found not only to have a very low activity, but was also found to produce a deleterious product by catalyzing a neomorphic reaction that converts α-KG to 2-hydroxyglutarate (2HG) by oxidizing NADPH to NADP^3^ (Fig. 1a). 2HG is an oncometabolite that inhibits α-KG-dependent dioxygenases, and the overall result of the mutation is DNA hypermethylation and epigenetic changes associated with neuronal proliferation^3, 4^. Structural analysis of the WT and mutant IDH1 indicate that R132 is a “gate-keeper” for the active site coordinating the hinge bending motion that defines the transition from the open to the closed (catalytically competent) conformation^1^. Mutations at R132 result in a reorganization of the interactions with the metal ion within the active site, disrupting ICA binding and favoring the conversion of α-KG to 2HG^3^.

Our previous work showed that trapping maltose binding protein (MBP) in its bound form using a conformation-specific antibody fragments (Fabs) increases the affinity of MBP for maltose. Additionally, the Fabs can restore the affinity of a binding pocket mutant of MBP by pushing the equilibrium towards the bound conformation^5^. We reasoned that an affinity reagent that recognizes and “locks-in” the WT IDH1 conformation would act as an activator of the mutant by shifting the equilibrium towards the active form (Fig. 1b).

## Results

We utilized a high-diversity (~10^10^) phage display library of humanized antibody fragments (Fabs)^6^ to select for Fabs that recognize the active conformation of IDH1. We carried out the phage display selection using the WT IDH1 in the presence of ICA and NADP. The selection also contained calcium, which locks the enzyme in an active conformation, while preventing turnover^1^. After four rounds of selection using the immobilized WT IDH1, an ELISA screen of 96 candidates revealed 9 unique Fab clones with the most favorable signal, which were named ID1 – ID9 (Supplementary Table 1). Since the Fabs can potentially recognize a number of different epitopes on IDH1, including those that are not conformationally different between the active and inactive forms, the Fab clones were screened individually to determine their influences on activity.

We measured the effect of the purified Fabs on the ability of the IDH1-R132H mutant to convert ICA to α-KG at a single, saturating concentration of ICA. At low concentrations (below 50 nM), the mutant activity is undetectable, and therefore, initial characterization was carried out using high concentration of the R132H mutant (500 nM) in the presence of 2–4 fold excess of the purified Fabs, whereas, the WT IDH1 was assayed at 5 nM enzyme concentration. Under these conditions, the ID7 Fab produced a ~30 fold rate enhancement of the R132H activity (Fig. 1c). In contrast, ID5 had a slight inhibitory effect, while the rest of the Fabs tested showed little to no effect on ICA conversion to α-KG (Fig. 1c). Detailed kinetic analysis reveled that excess ID7 enhances the ability of the mutant to convert ICA to α-KG (Fig. 1d) by lowering the *K*_M_ for ICA by 4.5 fold and accelerating the turnover rate, acting as both a K-type and a V-type activator with an overall *k*_cat_/*K*_M_ enhancement of 140 fold (Table 1). In contrast, ID7 has no effect on the rate of the neomorphic reaction (Supplementary Fig. 1).

**Table 1:** Thermodynamic and kinetic parameters for the enhancement of IDH1 mutant activity by ID7 Fab.

	*K*_M, ICA_ (μM)	*k*_cat_ (min^−1^)	*k*_cat_/*K*_M_ (min^−1^ μM^−1^)	*k*_cat_/*K*_M_ fold enhancement
WT IDH1	94	2.1 × 10^3^	22	—
R132H IDH1	790	0.44	5.6 × 10^−4^	—
R132H IDH1 + ID7	165	13	7.9 × 10^−2^	140
WT IDH1 + ID7	21	590	28	1.3

**Figure 2:**
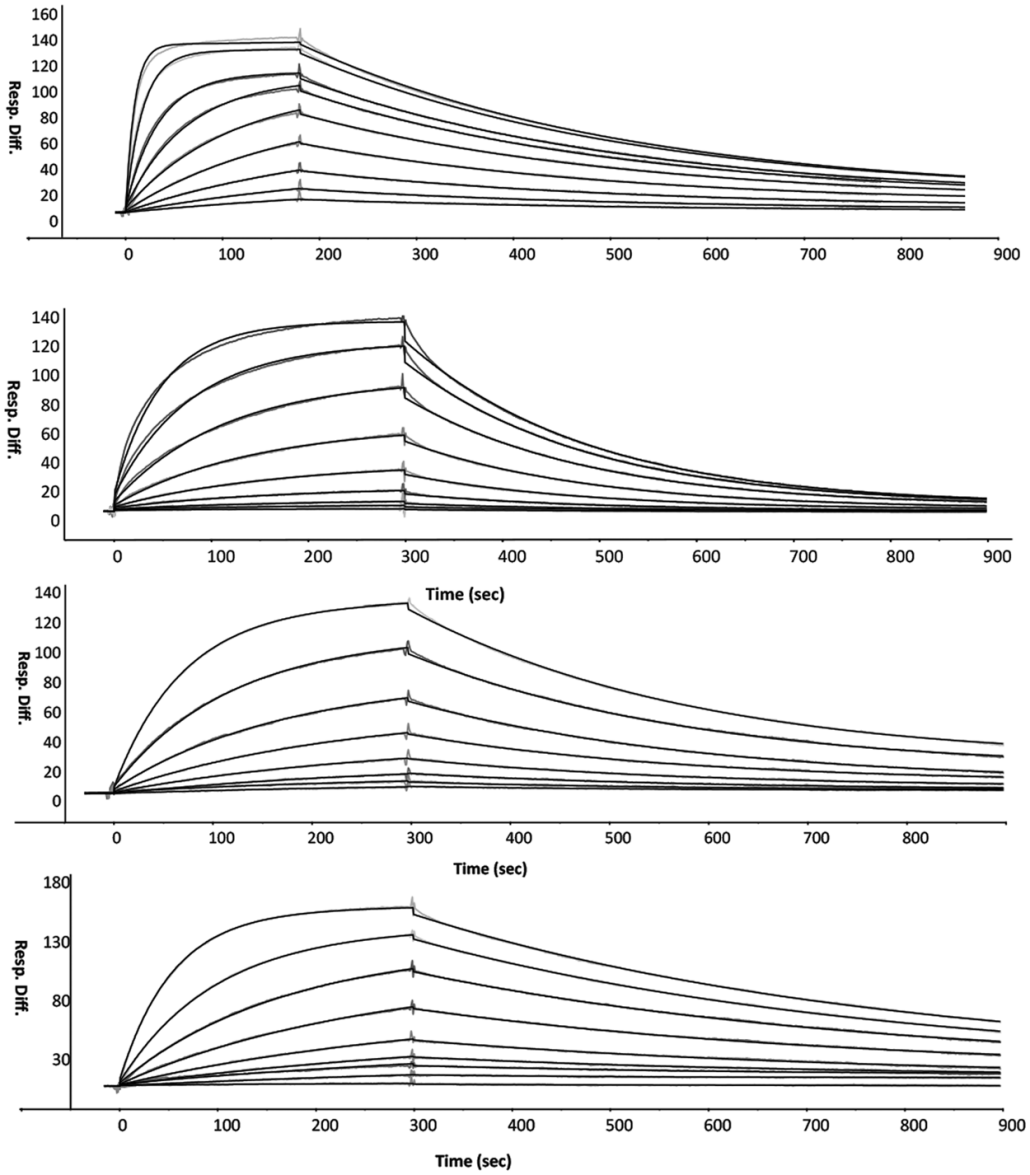
Binding kinetics for the interactions of Fabs ID7 and ID5 with the WT and mutant IDH1. (**a**) Interaction between ID7 and WT IDH1. (**b**) Interaction between ID7 and mutant IDH1. (**c**) Interaction between ID5 and WT IDH1. (**d**) Interaction between ID5 and mutant IDH1.

The ID7 Fab also increased the apparent affinity of the WT IDH1 for ICA as indicated by a 4.5 fold decrease in *K*_M_, similar to its effect on the mutant. However, it decreased the overall *V*_max_ of the enzyme (Fig. 1e). This is consistent with a model where the Fab favors the WT enzyme-substrate complex, promoting substrate binding in both WT and mutant, but may interfere with product release in the WT protein. Therefore, while ID7 decreases the *V*_max_ of the WT, it has an overall slight positive effect on the *k*_cat_/*K*_M_ value (Table 1). Surface plasmon resonance analysis of the affinity of ID7 for the WT and mutant IDH1 (Table 2) revealed that ID7 has a higher affinity for the WT IDH1 (Fig. 2a,b), with a ∆∆G of 1.9 kcal/mol. In contrast, ID5 slightly favors the mutant form of IDH1 (Fig. 2c,d). Epitope binning experiments suggest that ID7 and ID5 compete for different conformations of the enzyme. The signal intensity obtained from Fab ID5 binding is reduced by 80% when IDH1 is pre-saturated with ID7 compared with binding to IDH1 alone (Supplementary Fig. 2). ID7 is by far more conformationally-specific for the active form of IDH1 as indicated by the large difference in *K*_d_ values between the WT and mutant IDH1. As a result, ID7, which favors the WT enzyme conformation, can rescue the mutant by shifting the equilibrium towards the active form.

**Table 2:** Thermodynamic and kinetic parameters for the interactions between Fabs ID7 and ID5 with the WT and mutant IDH1.

Target	Fab	*k*_on_ (M^−1^s^−1^)	*k*_off_ (s^−1^)	*K*_d_ (nM)
WT IDH1	ID7	2.68 × 10^5^	2.77 × 10^−3^	10.3
Mutant IDH1	ID7	1.91 × 10^4^	5.16 × 10^−3^	271
WT IDH1	ID5	5.69 × 10^4^	3.14 × 10^−3^	55.1
Mutant IDH1	ID5	4.19 × 10^4^	1.96 × 10^−3^	46.7

**Figure 3:**
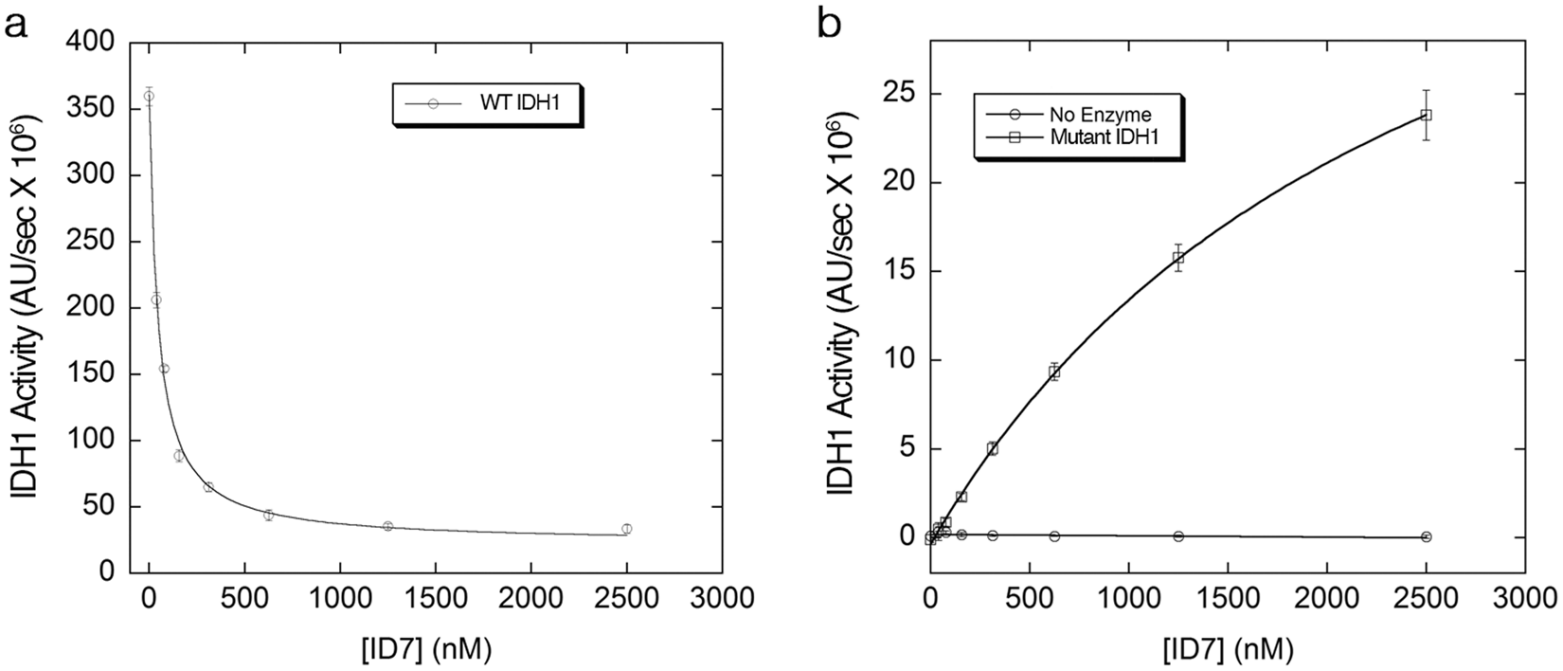
Concentration-dependent effects of ID7 on WT IDH1 and the R132H mutant. (**a**) Fab ID7 inhibits the WT enzyme in a concentration dependent manner with a *K*_i_ of 45 nM. (**b**) Fab ID7 activates the R132H mutant with a *K*_act_ of ~2 μM (squares), but has no intrinsic dehydrogenase activity (circles). Error bars reflect standard deviation.

We also determined the concentration-dependence of the ID7 Fab on modulation of protein function in both WT and mutant IDH1. As concentrations of the Fab were increased, the overall activity of the WT enzyme decreased with a *K*_i_ of 45 nM (Fig. 3a). In the case of the mutant enzyme, increasing concentration of the Fab resulted in an enhancement of enzyme activity with an activation constant (*K*_act_) of 2.5 μM (Fig. 3b). Altogether, the data show that ID7 favors the substrate-bound form of IDH1 as indicated by the enhancement in *K*_M_ values for both the WT and the mutant enzyme and the ability of the Fab to shift the mutant towards the active form in a concentration-dependent manner. This is consistent with classical allosteric activation models, where the effector acts by shifting the equilibrium from a less active to a more active conformation^7^.

## Discussion

The work shown here describes a general method to replicate the natural phenomenon of allostetic regulation of enzyme activity and provides a systematic approach for the development of “activator” molecules. We have previously shown that synthetic antigen binders and other engineered affinity reagents can be used to enhance the binding affinity of a protein for its ligand *in vivo* ^5^, modulate the specificity of a receptor for its cognate hormones *in situ* ^8^, rearrange cytoskeletal structure in mammalian cells^9^, and influence the substrate specificity of an enzyme^10^. Here, we show that a conformation-specific Fab can be used to rescue the function of a mutant enzyme associated with cancer.

While it is substantially more tenable to generate inhibitors of enzyme function^11^, the ability to systematically generate activators has so far been limited to large scale drug-screens^12–14^, enzymes with known allosteric sites that can be incorporated in the design process^15, 16^ or by pure serendipity^17^. Instead, here, we use a conformation-specific selection method to generate allosteric activators without targeting the active site or a known allosteric site. There are several advantages to this approach. First, *in vitro* phage display allows control over the conformation of the target protein, providing the ability to trap it in an active form by controlling the conditions during the selection process^5^. This is not easily attained by traditional immunization methods for the production of monoclonal antibodies, where the antigen is limited to the conditions in the animal’s bloodstream. Second, the selection is not based on enzyme activity, but rather on the enzyme conformation, and therefore, it can be adaptable to other systems where an enzyme assay is not suitable for a large-scale drug screen. Third, this method does not require detailed structural information about the target enzyme, but rather only about the conditions that favor its active form.

Ideally, an activator molecule would bring the level of the mutant to physiological levels. Here, the level of activation of the mutant IDH1 by ID7 is much lower than the level of the WT activity. However, unlike enzyme inhibitors, which must have a high level of efficacy to prevent “leaky” activity^11, 14^, it has been suggested that activator drugs are only required to induce a slight activation on the target enzyme, since often a low level of activity is sufficient to trigger a physiological output^18–20^. This is particularly important in relation to the vast number of enzyme deficiencies, where partial activation of the enzyme can have profound effects on the severity of the disease. Therefore, we believe that this strategy can be applied to thousands of disorders where loss-of-function mutations disrupt protein conformation, ligand/substrate binding or protein-protein interactions.

## Methods

### IDH1 expression and purification

Human IDH1 plasmid was purchased from Origene. The WT and R132H constructs were cloned into pET25b(+) using NheI and NdeI restriction sites. Both the WT and mutant were expressed in BL21(DE3) *E. coli* cells induced with 1 mM IPTG at 20 °C overnight, and purified using an IMAC column, followed by size exclusion on a Superdex 200 (16/60) column (GE) in 50 mM Sodium Phosphate, 150 mM NaCl, pH 7.0.

### Phage selection

The selection was carried out as described^5, 21^. Briefly, biotinylated WT-IDH1 was immobilized on streptavidin magnetic beads and mixed with 1 mL of phage library in TBST containing 5% BSA, 30 mM ICA, 1 mM NADP, 40 mM CaCl_2_. The beads were washed 3 times with the same buffer, then used to infect XL-1 Blue cells in mid-log phase. Three additional rounds were carried out, each with decreasing protein concentrations (Round 1: 200 nM; Round 2: 100 nM, Rounds 3 & 4: 50 nM).

### Characterization of Fab clones

192 clones were tested for the ability to bind specifically to WT-IDH1 in the presence of ICA, NADP and Calcium using a competitive phage ELISA. Successful clones are those which produced a high ELISA signal in wells where IDH1 is immobilized and a low signal where free IDH1 is present as a competitor^21^. Clones that showed a high ELISA signal and a low competition ratio were selected for further characterization. First, DNA sequencing was used to determine the number of unique clones, then, a stop codon was inserted between the Fab and the phage pIII gene using Kunkle mutagenesis. Fabs were expressed using phosphate-depleted CRAP media as described^21^, and purified using affinity chromatography on protein G or protein A resin, followed by ion exchange on an Hi-trap SP column (GE).

### Enzyme Assays

All enzymatic assays were carried out in 96-well clear-bottom plates with a total volume of 100 μL per reaction. The reactions were carried out in 30 mM TRIS pH 7.4, 0.1 mM NADP, 2 mM MnSO_4_ in the presence or absence of Fab, with the WT-IDH1 or the R132H mutant. Isocitrate was added to the wells using a multichannel pipette to start the reaction. The change in absorbance at 340 nm was recorded over time to monitor the production of NADPH, and the slope was used to determine initial velocities. An extinction coefficient of 6,270 M^−1^cm^−1^ for NADPH was used to convert the changes in absorbance to enzyme rates. Kaliedagraph was used for plotting and data fitting.

### Binding Analysis

Binding kinetics of WT and mutant IDH1 with Fabs ID5 and ID7 (analyte) were performed at 20 °C using a BIACORE 3000 (GE Healthcare). Pure, monodispersed, His_10_-tagged WT and mutant IDH1 (ligand) were immobilized onto an NTA sensor chip. For each kinetic experiment, two-fold dilution series of Fabs were injected at a flow rate of 30 μl/min. All conditions were tested for nine different Fab concentrations. To determine kinetic rate constants, all data sets were fit to a simple 1:1 interaction model using BiaEvaluation Software (GE).

For the epitope binning experiments, 20 nM His_10_-tagged wild type IDH1 was immobilized on an NTA chip at 20 °C. 800 nM ID7 was injected for 100 sec at a flow rate of 30 ul/min to saturate the epitope. After saturating the target on the chip with ID7, a mixture of 800 nM ID7 and 800 nM ID5 was injected. 800 nM ID7 was added in the second injection as well to make sure that the epitope recognized by the first Fab remained fully saturated during the interaction of IDH1 with the second Fab. Control experiment was done using ID7 alone in a second injection.

## Electronic supplementary material

Supplementary Information
