# Supplementary material for: Targeted rescue of cancer-associated IDH1 mutant activity using an engineered synthetic antibody
Source: Sci Rep. 2017 Apr 3;7:556. doi: 10.1038/s41598-017-00728-1 (PMC5429742; doi:10.1038/s41598-017-00728-1)
Supplement: Supplementary file 1 — Supplementary Information [file 41598_2017_728_MOESM1_ESM.pdf]

Supplementary Information

Targeted rescue of cancer-associated IDH1 mutant activity using an engineered synthetic antibody

Shahir S. Rizk[1*], Somnath Mukherjee[2], Akiko Koide[3], Shohei Koide[2,3] and Anthony A. Kossiakoff[2]

[1]Department of Chemistry and Biochemistry, Indiana University South Bend, Department of Biochemistry and Molecular Biology, Indiana University School of Medicine, South Bend, Indiana

[2]Department of Biochemistry and Molecular Biology, University of Chicago, Chicago, Illinois

[3]Biochemistry and Molecular Pharmacology Department, Alexandria Center for Life Sciences, New York University, New York, New York

[*]Corresponding Author

Supplementary Table 1: Sequence of CDRs of Fab clones

Fab	Loop Sequence			
	L3	H1	H2	H3
**ID1**	SSSSLI	LYYSS	SISPYSGYTS	DYWWWWFEYVAM
**ID2**	QYWFLI	VYYSS	SIYSSSGYTS	YWYSWAM
**ID3**	YGSSPLI	VSYSS	SIYSSYGYTY	HYYSHWYGGVYAM
**ID4**	SQNYLI	ISSSS	SIYSYYGSTY	GSVWYWWWPGI
**ID5**	YGQSSLV	VYSSY	SISPYSGYTY	SGSYLAL
**ID6**	WGWSSYLI	FYYSS	YIYPYSGSTS	FPWYRYYWAM
**ID7**	APYGWLI	IYSSS	SIYPYSGYTS	GWYGWNTL
**ID8**	YGYMLV	IYYYS	SIYSSYGSTY	WYVSSWYWWQWAM
**ID9**	RYYWLI	ISYSS	SIYSSSGYTY	SSWSGM

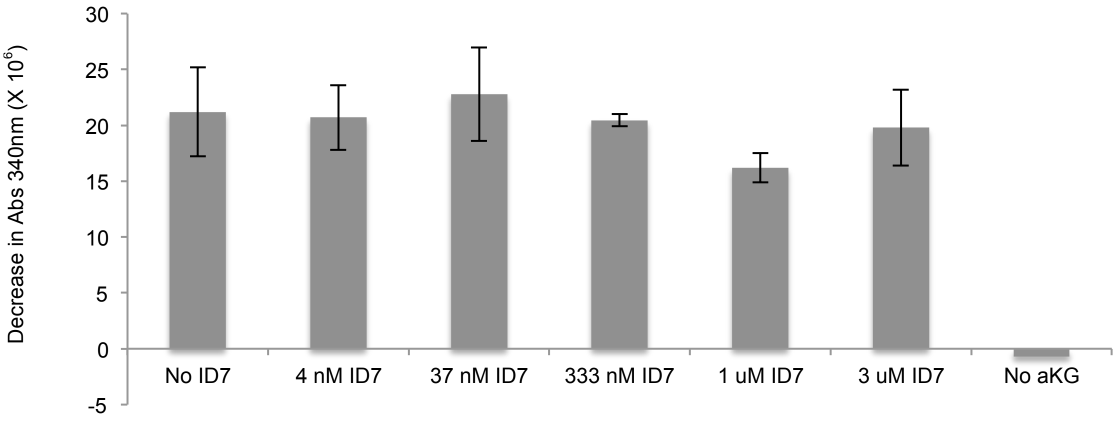

Supplementary Figure 1: The effect of ID7 on the neomophic activity of the IDH1 R132H mutant. The neomorphic reaction of the IDH1 mutant was detected by monitoring the decrease in absorbance at 340nm, indicating the conversion of NADPH to NADP and αKG to 2HG. The rate was measured using 500nM IDH1 R132H, 100μM NADPH, 1mM αKG. No change in the rate was observed as a result of addition of increasing amounts of Fab ID7.

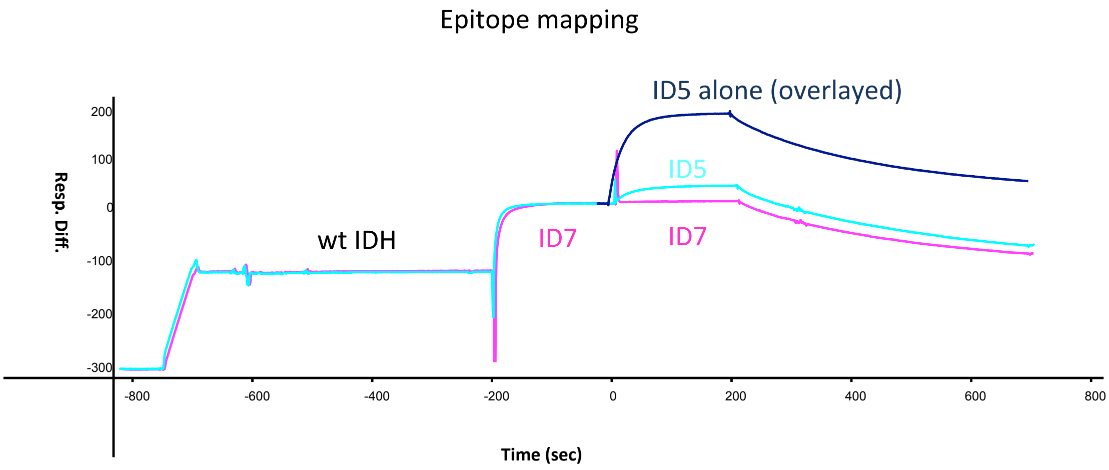

Supplementary Figure 2: Epitope binning of ID7 and ID5 for WT IDH1. When a mixture of ID5 and ID7 was injected on immobilized WT IDH1 saturated with ID7 (cyan), there was a small increase in signal (~ 30 RU) which shows that ID5 binds in presence of ID7. However, the signal intensity is 20% of the signal obtained when ID5 was injected alone (dark blue) on immobilized WT IDH1 (without pre-saturation it with ID7). There is no increase in signal when ID7 is injected after saturating the surface with ID7 (pink).
